# Tailoring Matrix Toughness for High-Performance Composites in Cryogenic Applications

**DOI:** 10.3390/polym18151864

**Published:** 2026-07-29

**Authors:** Helena C. Teixeira, Renata C. Oliveira, Andreia Araújo, Joana F. Guedes

**Affiliations:** 1 Institute of Science and Innovation in Mechanical and Industrial Engineering (INEGI), R. Dr. Roberto Frias 400, 4200-465 Porto, Portugal; hteixeira@inegi.up.pt (H.C.T.); rcoliveira@inegi.up.pt (R.C.O.); 2 Associated Laboratory for Energy, Transports and Aerospace (LAETA), R. Dr. Roberto Frias 400, 4200-465 Porto, Portugal

**Keywords:** epoxy toughening, fracture toughness, carbon fibre-reinforced polymer (CFRP), cryogenic performance

## Abstract

The rapid expansion of space exploration has increased the demand for lightweight structural materials capable of maintaining performance under extreme thermal conditions. Carbon fibre-reinforced polymers (CFRPs) offer high specific strength and low density; however, their application in cryogenic environments remains challenging due to the brittleness of epoxy matrices, which are susceptible to cracking at low temperatures and under thermal cycling. In this work, two strategies were investigated to improve the damage tolerance of epoxy nanocomposites: (i) the use of a biscitraconimide-based (BCI) resin and (ii) the incorporation of methyl methacrylate–butadiene–styrene (MBS) core–shell particles. Low additive contents were evaluated to identify formulations compatible with prepreg manufacturing. The incorporation of 2 wt.% of MBS core–shell particles significantly improved the impact resistance of the nanocomposites and was selected for CFRP laminate production. When applied to CFRPs, the modified matrix maintained the overall tensile behaviour while increasing the interlaminar fracture toughness by 102% and 122% at room (RT) and cryogenic temperatures (CT), respectively. These findings demonstrate that matrix modification using low-content toughening is an effective strategy to enhance the cryogenic performance of CFRPs, contributing to the development of lighter and more resilient composite structures for next-generation space systems.

## 1. Introduction

The growth of space exploration has increased the demand for advanced materials capable of reducing structural weight, thereby lowering launch costs and increasing payload capacity [1]. Therefore, the replacement of conventional materials, such as metals, by lightweight alternatives, particularly fibre-reinforced polymers (FRPs), has emerged as a major research focus. FRPs play a key role in high-performance applications across the aerospace, automotive, and energy sectors [2,3]. These materials offer several advantages, including low density, corrosion resistance, good fatigue performance, design flexibility, and a high strength-to-weight ratio, making them excellent candidates for weight-critical structures [4,5]. However, space applications impose severe thermal conditions, as launch vehicles and spacecraft are exposed to repeated thermal cycles and cryogenic temperatures (CTs). Under these conditions, the mechanical behaviour and failure mechanisms of FRPs can differ substantially from those observed at room temperature (RT) [5].

In composite materials, the polymer matrix is responsible not only for transferring loads between the fibres, but also for maintaining the structural integrity and ensuring the long-term durability of the resulting structures [4]. Thermosetting polymers are widely used as matrices owing to their ease of processability and appealing properties. Among them, epoxy matrices exhibit excellent mechanical performance, thermal stability and chemical resistance [6,7]. However, they are inherently brittle, displaying relatively low fracture toughness and limited resistance to crack propagation [8,9,10]. These limitations restrict the application of carbon fibre-reinforced polymer (CFRP) composites with epoxy resin in cryogenic environments [10,11,12] where microcrack formation may occur, ultimately leading to the failure of the structure [13]. Thus, improving the toughness of epoxy matrices is essential for developing composites suitable for cryogenic applications.

One of the most effective strategies to improve fracture toughness is matrix modification by incorporating reinforcing or toughening agents, including rigid nanoparticles (e.g., carbon nanotubes, graphene nanoplatelets, graphene oxide), thermoplastic or flexible polymers, block copolymers, and rubber particles [3,11,14]. Several studies have reported promising results [13,15,16,17].

In this context, biscitraconimide (BCI)-based systems have been widely investigated as epoxy modifiers under both RT and CT. Liu et al. [9] investigated the incorporation of biscitraconimide resin at different concentrations into an epoxy resin to improve tensile, bending, and impact strengths, as well as fracture toughness at both temperatures. The authors reported that the addition of 30 wt.% BCI increased the fracture toughness by 114.1% at RT and 65.9% at −196 °C compared with the unmodified epoxy [9]. In a subsequent study, Liu et al. [12] combined 4 wt.% of a reactive phosphorus-/nitrogen-containing fire retardant (BSEA) with 25 wt.% BCI resin to improve the fracture toughness and impact performance of the nanocomposite. This formulation increased fracture toughness by 125.17% at RT and 81.10% at CT relative to neat epoxy.

In addition to resin-based modifications, core–shell particles have emerged as promising toughening agents for epoxy systems. Deng et al. [3] investigated the effect of incorporating 3 wt.% of an organic core–inorganic shell nanoparticle, poly(butyl acrylate)@SiO_2_ (PBA@SiO_2_), and reported an 85.6% increase in impact strength compared with unmodified epoxy. Similar improvements have been observed with other core–shell systems. For example, Giannakopoulos et al. [18] concluded that the addition of 15 wt.% of a 100 nm core–shell rubber (CSR) particle to the epoxy increases the fracture energy from 77 to 840 J·m^−2^ at RT, highlighting their potential as effective toughening agents and suggesting that their performance under cryogenic conditions merits further investigation. Additionally, Beylergil et al. [19] evaluated the effect of adding different concentrations of Clearstrength^®^ XT100 to the CFRP. It was concluded that adding 3, 5 and 7 wt.% of the methylmethacrylate–butadiene–styrene (MBS) core–shell particle increases the *G_IC_* by 70%, 91% and 177%, respectively, in relation to unmodified CFRP [19].

Other particle types have also shown promising results in improving fracture toughness. Shirodkar et al. [20] produced nanocomposites with different concentrations of graphene nanoplatelets (GNPs), carbon nanotubes (CNTs), and hybrid GNP/CNT systems to evaluate potential synergistic effects. The addition of 0.5 wt.% of GNPs, 0.5 wt.% CNTs, and a hybrid mixture of 0.25 wt.% of each nanofiller resulted in increases in critical stress intensity factor (*K_IC_*) of 110%, 118%, and 106%, respectively, compared to the neat epoxy. Likewise, Zotti et al. [21] dispersed 0.5 wt.% of GNPs into an epoxy matrix, resulting in increases in fracture toughness of 14.7% and 13.2% at RT and CT, respectively.

Although matrix modification has proven to be an effective and promising strategy for improving fracture toughness, the incorporation of nanomaterials and thermoplastic modifiers often increases the viscosity of the epoxy resin, which is a limiting factor for their application in the pre-impregnation process, highly used in high-performance applications [11,22]. Furthermore, achieving a homogeneous dispersion of the different additives in the epoxy resin remains a critical challenge, as particle agglomeration can significantly reduce the effectiveness of the toughening agent [7,11]. Consequently, careful control of additive content, viscosity, and dispersion quality is essential for identifying formulations that combine improved mechanical performance with manufacturing feasibility.

The main objective of this work is to enhance the toughness of CFRP laminates by adding lower loadings of toughening agents. To achieve this goal, two promising particles at low contents were studied: a resin based on BCI and an MBS core–shell particle. In the first stage of the study, epoxy resin was modified with varying concentrations of the particles mentioned (nanocomposites). The most effective additive and its optimal concentration were selected to produce carbon fibre pre-impregnated materials, based on dispersion, processability and impact properties. Finally, the influence of matrix modification on the mechanical properties of CFRP was evaluated under both RT and CT conditions. As mentioned, low additive contents were deliberately studied, 2 wt.% and 4 wt.%, to achieve a good compromise between the processability of the resin, a critical constraint for the pre-impregnation process, and improvements in its toughness and mechanical properties.

## 2. Materials and Methods

### 2.1. Materials

In this study, a 24 K carbon fibre roving (Tenax™-E STS40 E23 24 K 1600 tex), supplied from Teijin (Tokyo, Japan), was used as the reinforcement. The matrix consisted of a pre-impregnation system with a chemical B-stage, comprising Araldite^®^ LY556, Aradur^®^ 1571, Accelerator 1573, and Hardener XB3403, in a weight ratio of 100:23:5:12, supplied by Huntsman Chemicals (The Woodlands, TX, USA).

Two different toughening additives were selected to improve the composite material’s toughness. A BCI-based resin (Homide 400), kindly provided by HOS-Technik (St. Stefan im Lavanttal, Austria), was used as a resolidified melt with a softening range of 70 °C and 120 °C. In addition, MBS core–shell particles (Clearstrength^®^ XT100), also kindly provided by Arkema (Puteaux, France), were used as a white powder with a bulk density of 0.3 g·cm^−3^. The average size of the powder particles is approximately 200 μm, while the primary MBS core–shell particles have an average diameter below 200 nm.

### 2.2. Manufacturing Process

#### 2.2.1. Matrix Modification and Production of Nanocomposites

Two additive loadings (2 wt.% and 4 wt.%) were selected to evaluate their effect on the nanocomposites. These concentrations were chosen as a starting point based on a literature review [3,14,16,19] and supplier consultations, while also considering that low additive content is desirable to preserve the resin’s processability, particularly its viscosity, which is critical for the production of pre-impregnated materials. Additionally, this range allowed assessment of whether significant toughness improvements could be achieved at low additive contents, since BCI resin is typically used at higher loadings in prior studies [9]. Table 1 summarises the selected particles, concentrations and the incorporation method used.

The BCI resin was first mixed with the epoxy resin Araldite^®^ LY556 at 1250 rpm in a silicone oil heating bath at 100 °C to ensure homogeneous temperature. The mixture was then cooled to room temperature to minimise premature curing, after which the hardeners were added and mixed for 30 min at 300 rpm. A similar procedure was followed for the Clearstrength^®^ XT100 particles, although a lower mixing speed of 750 rpm was used. All dispersions were subsequently degassed and cured at 120 °C for 2 h.

#### 2.2.2. Production of CFRP Laminates

Pre-impregnated carbon fibre/epoxy resin materials were produced using laboratory-scale pre-impregnation equipment. Several processing parameters, including the pitch, line speed, and fibre pre-tension, were optimised to control the prepreg resin content. During manufacturing, carbon fibres were passed through a resin bath and a series of rollers, with excess resin removed by nip rolls, thereby controlling the resin-to-fibre ratio. The resin content of the prepregs was determined by matrix digestion in acetone, according to the ASTM D3529 standard [23]. Additionally, the thickness of each ply was experimentally determined by stacking ten plies, consolidating them in an autoclave, and measuring the total laminate thickness to calculate the average ply thickness.

**Figure 1:**
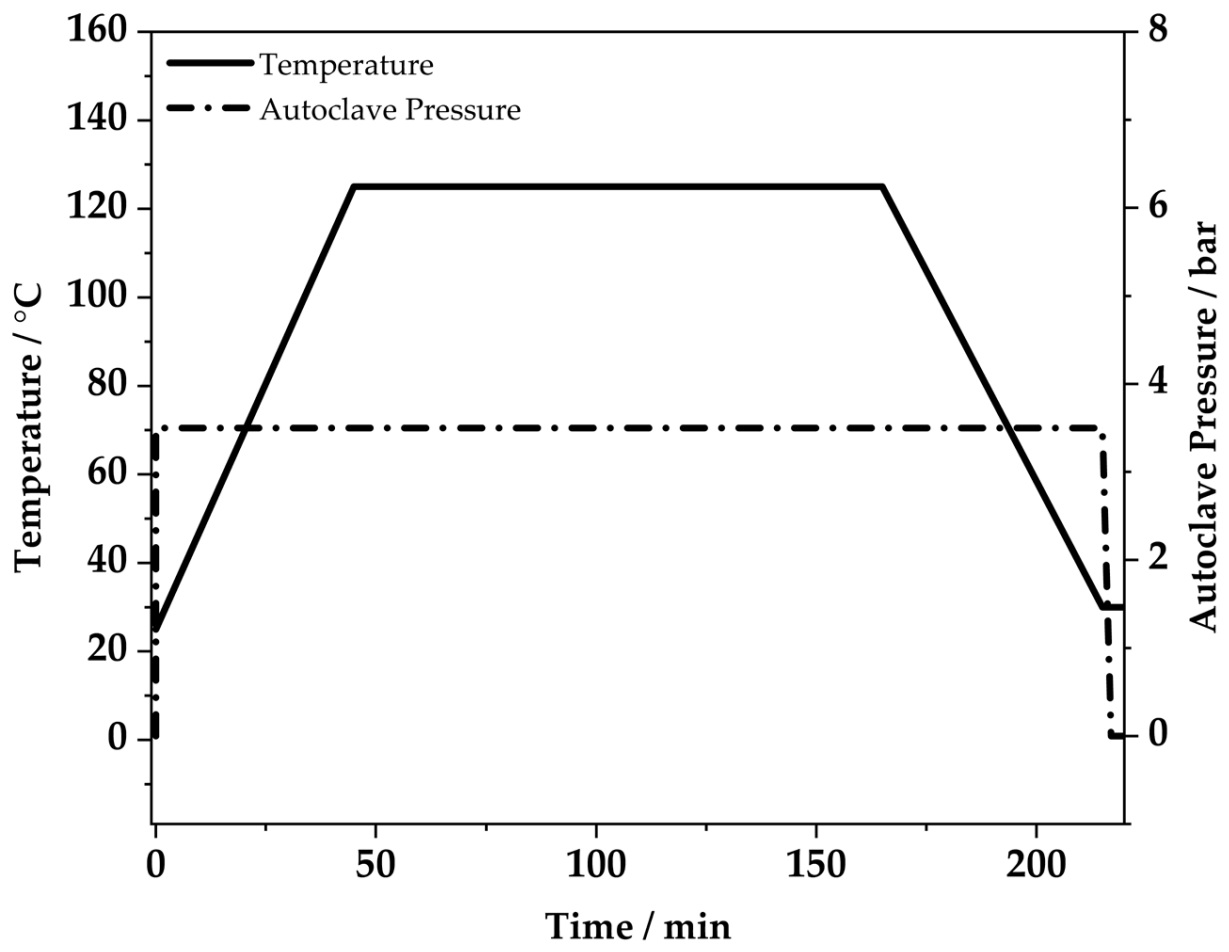
Typical cure cycle of Araldite^®^ LY556 pre-impregnation system.

Finally, CFRP laminates of different thicknesses, in accordance with the characterisation test plan, were produced by stacking the required number of prepreg plies. The materials were cured in an autoclave at 120 °C for 2 h with a pressure of 3.5 bar, as presented in Figure 1.

### 2.3. Characterisation and Testing Procedures

#### 2.3.1. Nanocomposites

Scanning electron microscopy (SEM) analyses were conducted using a High Resolution (Schottky) Environmental Scanning Electron Microscope with X-ray microanalysis and Electron Backscattered Diffraction Analysis, FEI Quanta 400 FEG ESEM/EDAX Genesis X4M (Thermo Fisher Scientific, Waltham, MA, USA). The samples were coated by sputtering with an Au/Pd thin film using the SPI Module Sputter Coater Equipment (Structure Probe, Inc. (SPI Supplies), West Chester, PA, USA).

Differential scanning calorimetry (DSC) experiments were performed using a Q20^®^ model calorimeter from TA Instruments^®^ (New Castle, DE, USA) to assess whether the curing reactions were complete and evaluate the thermal transitions of the nanocomposites. The experiments were performed in accordance with ISO 11357 [24]. The samples were heated from 30 °C to 250 °C at a constant rate of 10 °C·min^−1^, and then cooled down to 30 °C before being heated again to 250 °C, under a nitrogen (N_2_) flow rate of 50 mL·min^−1^. Each sample was tested three times to ensure the reproducibility of the results.

Charpy impact tests were conducted on unnotched samples measuring 80 mm × 10 mm × 4 mm in accordance with ISO 179 [25]. The samples were subjected to an impact of a 5 J pendulum, with intrinsic loss being verified before each set of samples. The Charpy impact test was performed at RT as a standard comparative assessment of the toughening effect of added particles on the material’s bulk impact resistance. Ten replicate tests were conducted for each formulation. To determine the Charpy impact strength of unnotched specimens, *a_cU_* (kJ·m^−2^), Equation (1) was used. Additionally, to evaluate the statistical significance of the results, a one-way analysis of variance (ANOVA) was conducted to compare the 5 studied groups, followed by Student’s *t*-tests for pairwise comparisons. A 95% confidence interval was used, with *p*-values < 0.05 considered statistically significant. All analyses were conducted using Data Analysis ToolPak in Microsoft^®^ Excel^®^ for Microsoft 365 MSO (Version 2607 Build 16.0.20228.20110) 64-bit.

(1) $$a_{cU}=\frac{E_{c}}{h\times b}\times 10^{3}$$

where *Ec* is the corrected impact energy absorbed by breaking the specimen (J), *h* is the specimen thickness (mm), and *b* is the specimen width (mm).

The processability of the dispersion was evaluated by rheological testing with a Discovery HR-1 rheometer from TA Instruments^®^ (New Castle, DE, USA) equipped with a 25 mm aluminium parallel-plate geometry. A flow-ramp method was applied to the mixture (resin with hardeners) at 25 °C, covering a shear-rate range of 0.1 to 100 s^−1^. Measurements were carried out both immediately after mixing with the hardeners (t = 0 min) and after 2 h (t = 120 min), which represents the typical processing window employed in the laboratory-scale pre-impregnation equipment used in this study.

#### 2.3.2. CFRP

The density of the CFRP was measured according to Archimedes’ Principle following Method A of ASTM D 792 standard [26], and the fibre volume fraction (FVF) and void content were determined through a burn-off test following ASTM 3171–G standard [27]. Additionally, thermogravimetric analysis (TGA) was used to evaluate the behaviour of the additives as a function of temperature. These tests were conducted with an STA 449 F3 Jupiter^®^ NETZSCH (Selb, Germany) equipment under an N_2_ atmosphere. Two sets of tests were conducted. First, the samples were heated from 25 °C to 800 °C with a heating rate of 10 °C·min^−1^ to evaluate the overall behaviour of the Clearstrength^®^ XT100 particles. Then, another test was performed under the conditions of the matrix burn-off test used for FVF determination, i.e., the samples were heated from 25 °C to 425 °C at 10 °C·min^−1^, followed by an isotherm at 425 °C for 6 h.

Longitudinal (0°) and transversal (90°) tensile tests were carried out according to the ASTM 3039 standard [28]. Specimens measuring 15 mm in width, 1 mm in thickness, and a gauge length of 138 mm were used for the tests in the fibre direction. Further, 25 mm wide, 2 mm thick specimens and a 125 mm gauge length were used for the tests transverse to the fibre direction. Both tests were conducted at a loading rate of 2 mm·min^−1^. At least five replicate tests were performed for each material at both RT and CT. For cryogenic performance, the samples underwent 15 cycles of immersion in liquid nitrogen (LN_2_) for 3 min each, with rest periods at RT in between. The parameters were chosen based on the existing literature, since several authors have reported that 3 min may be sufficient to cool the specimens to −196.15 °C, and different numbers of cycles have already been tested [29,30,31,32].

To determine the interlaminar fracture toughness of the laminates, the Mode I Double Cantilever Beam (DCB) test was conducted according to the ASTM D5528 standard [33]. The samples were tested at RT and CT. As in the tensile tests, to assess cryogenic performance, the samples were subjected to 15 cycles of immersion in LN_2_ for 3 min each, with rest periods at RT in between. Samples measuring 20 mm in width, 215 mm in length and 4 mm in thickness were used. A polytetrafluoroethylene (PTFE) film was inserted during the lamination to form an initiation site for the delamination. In these tests, the load was applied perpendicular to the crack plane with a crosshead speed of 2.5 mm·min^−1^. The mode I interlaminar fracture toughness, *G_IC_* (kJ·m^−2^), was calculated using the Modified Beam Theory (MBT) method, which yields conservative values via Equation (2) [34]. The point of deviation from the linearity of the load–displacement curve was selected as the critical point corresponding to the initiation of delamination.

(2) $$G_{IC}=\frac{3P\delta }{2b(a+\left|∆\right|)}$$

where *P* is the applied load (N), *δ* is the load-point displacement (mm), *b* is the specimen width (mm), *a* is the delamination length (mm), and Δ is the crack length correction factor, calculated experimentally by plotting the cube root of compliance C^1/3^ against the crack length *a*.

For all mechanical testing results, Student’s *t*-test was performed to evaluate differences between two groups, assuming equal variances, at the 95% confidence level. Results were considered statistically significant if *p*-values were less than 0.05. All analyses were conducted using the Data Analysis ToolPak in Microsoft^®^ Excel^®^ for Microsoft 365 MSO (Version 2607 Build 16.0.20228.20110) 64-bit.

## 3. Results and Discussion

### 3.1. Nanocomposites

**Figure 2:**
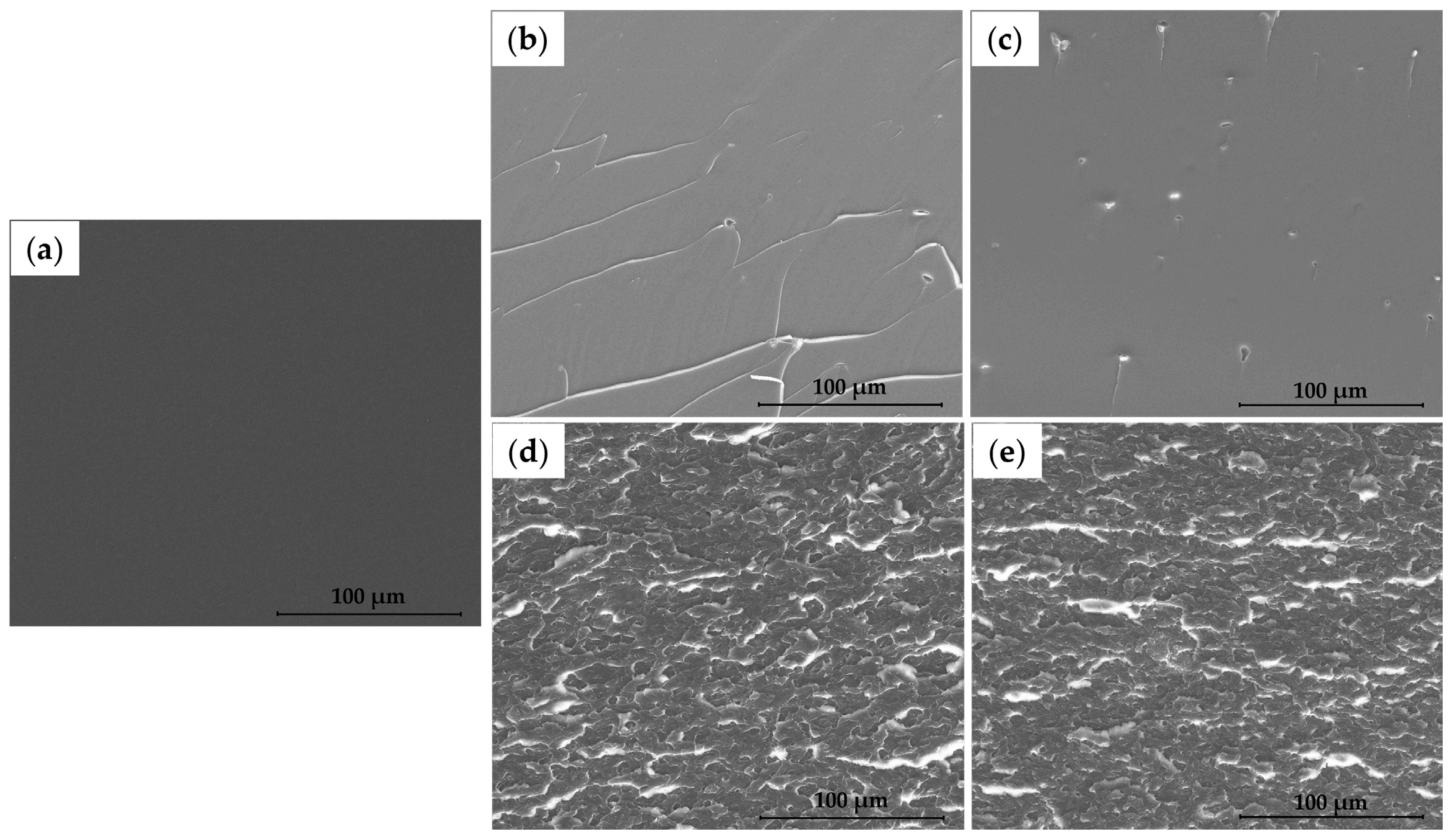
SEM images of (**a**) neat epoxy resin LY556, (**b**) LY556_2BCI, (**c**) LY556_4BCI, (**d**) LY556_2XT100, (**e**) LY556_4XT100, all with a 1000× magnification.

SEM analysis provides detailed information about the surface morphology, composition, and structure of the studied nanocomposites at the micro and nanoscale (Figure 2). At the same time, it allows the identification of toughness mechanisms.

**Figure 3:**
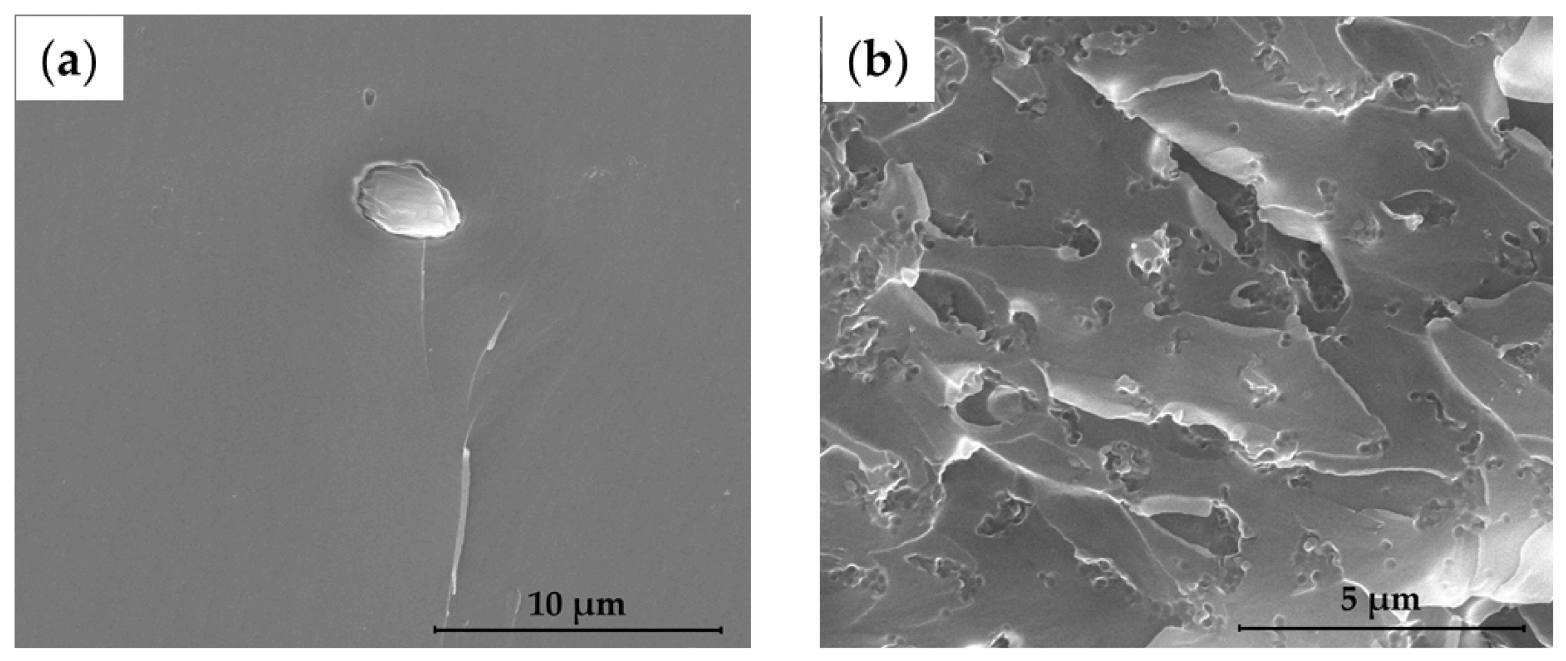
SEM detailed images: (**a**) BCI nanocomposites with some non-incorporated parts, with a magnification of 10,000× and (**b**) Clearstrength^®^ XT100 particles incorporated into the epoxy resin, with a magnification of 20,000×.

The neat epoxy (Figure 2a) presented a single phase with a clean fracture, suggesting that, as expected, its behaviour is brittle. The BCI samples (Figure 2b,c) revealed several areas that might indicate less effective incorporation of the BCI resin, which is composed of granules of varying sizes, as detailed in the granule image presented in Figure 3a. The BCI-modified samples exhibited a fracture surface characterised by crack deflection and phase-separated domains, suggesting that toughening was governed primarily by crack-path deviation and interfacial energy absorption [9,12]. On the other hand, the nanocomposites with Clearstrength^®^ XT100 (Figure 2d,e) show a uniform distribution of small spherical particles within the sample, suggesting effective dispersion. A rough texture was also observed in the fracture area, suggesting higher fracture strength and, consequently, an increase in fracture toughness. In this case, the rough fracture surface indicates particle cavitation and extensive matrix deformation, suggesting that energy was dissipated primarily through shear yielding of the surrounding matrix [35,36]. Further details are presented in Figure 3b, where spherical features with diameters in the range of 150–200 nm are observed, consistent with the reported size of the MBS core–shell particles.

DSC measures the heat flow associated with phase transitions, such as melting, crystallisation, and glass transitions, as a function of temperature, allowing the evaluation of the thermal stability of the nanocomposites. As mentioned previously, two ramps were applied in the DSC analysis: the first assessed whether residual heat remained in the sample, enabling evaluation of the degree of cure, and the second removed the thermal history, allowing determination of the material’s glass transition temperature (*T_g_*).

Table 2 lists the *T_g_* values for all the nanocomposites from the second heating ramp. The unmodified epoxy resin’s *T_g_* is in accordance with the supplier’s datasheet, falling within the range of 110–115 °C. The addition of various particles does not significantly affect this value, with variations of around 3–5%. Also, these variations are often attributed to the equipment’s sensitivity and to the sample preparation methods used. This behaviour is crucial for developing toughened epoxy resins, as it suggests that incorporating these additives enhances the material’s toughness while preserving its *T_g_* and thermal properties.

**Figure 4:**
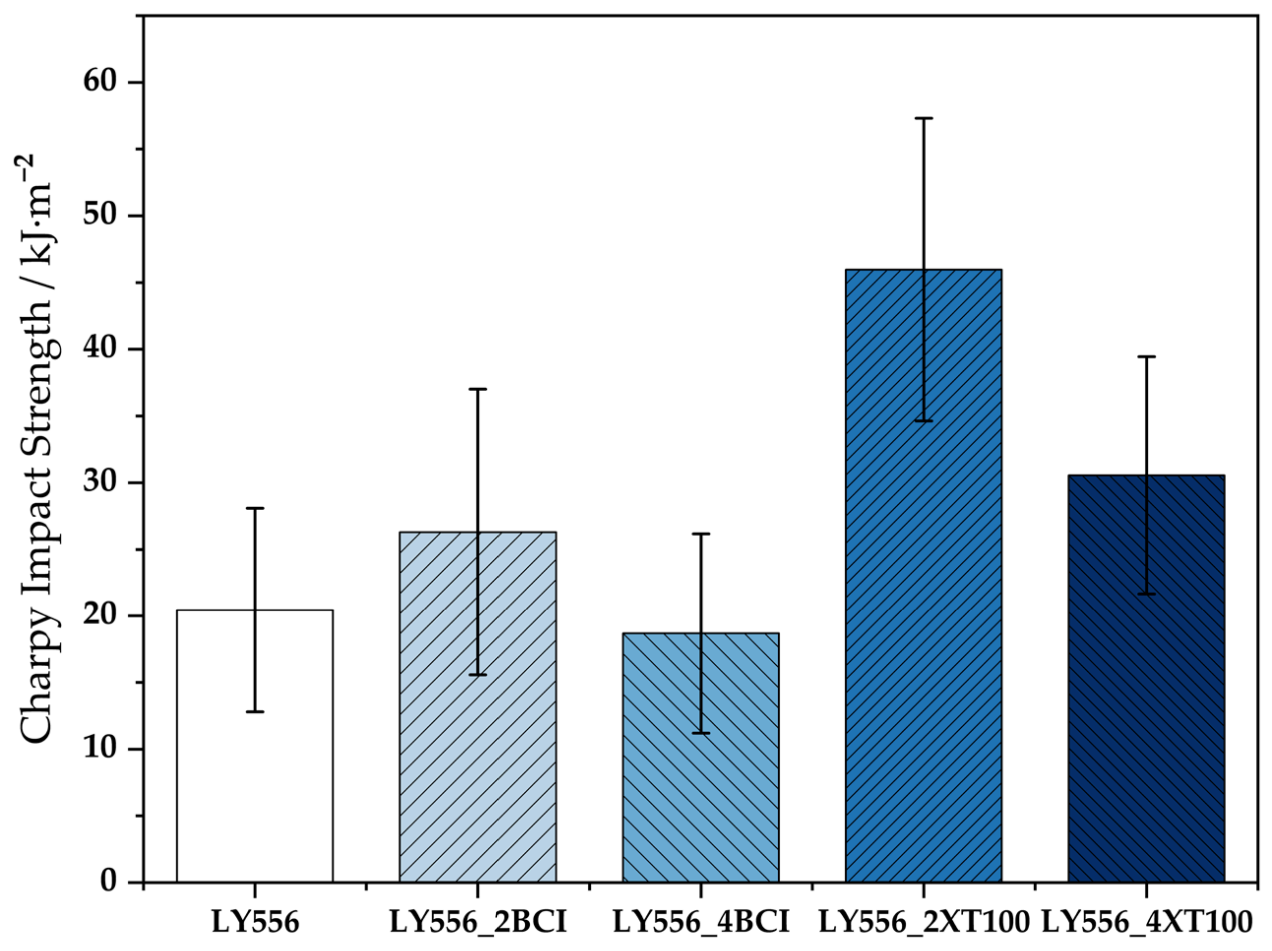
Charpy impact test results of the nanocomposites.

Charpy impact tests were conducted to assess the impact resistance and energy absorption capability of the nanocomposite specimens. Generally, higher absorbed energy indicates improved resistance to crack initiation and propagation, reflecting enhanced toughness and structural integrity under impact loading. Figure 4 shows the Charpy impact test results of the nanocomposites at RT. A one-way ANOVA at a 95% confidence level revealed a statistically significant effect of additive type or concentration on the Charpy impact strength of the nanocomposites: F(4,36) = 9.995, *p* = 0.000015.

The addition of BCI resin at a concentration of 2 wt.% resulted in a 29% increase in impact strength; however, this change was not statistically significant (*p* = 0.210). Similarly, the addition of 4 wt.% of the same additive did not produce a significant change (*p* = 0.660). These results may be attributed to certain parts of the BCI resin that were not fully incorporated into the epoxy resin, as observed in the SEM images (Figure 2b,c and Figure 3a). It is important to note that some previous studies have reported positive outcomes with higher BCI concentrations, such as 30 wt.%, indicating that saturation might not be the cause [9].

In contrast, the addition of 2 wt.% of Clearstrength^®^ XT100 particles resulted in the greatest improvement in Charpy impact strength with a significant increase (*p* = 0.00012) of 125% compared with the unmodified epoxy resin. This remarkable improvement demonstrates the effectiveness of Clearstrength^®^ XT100 as an impact modifier for epoxy systems. The enhanced impact performance is likely associated with the activation of toughening mechanisms, including particle cavitation and shear yielding of the surrounding matrix, which increase the energy required for crack initiation and propagation.

**Figure 5:**
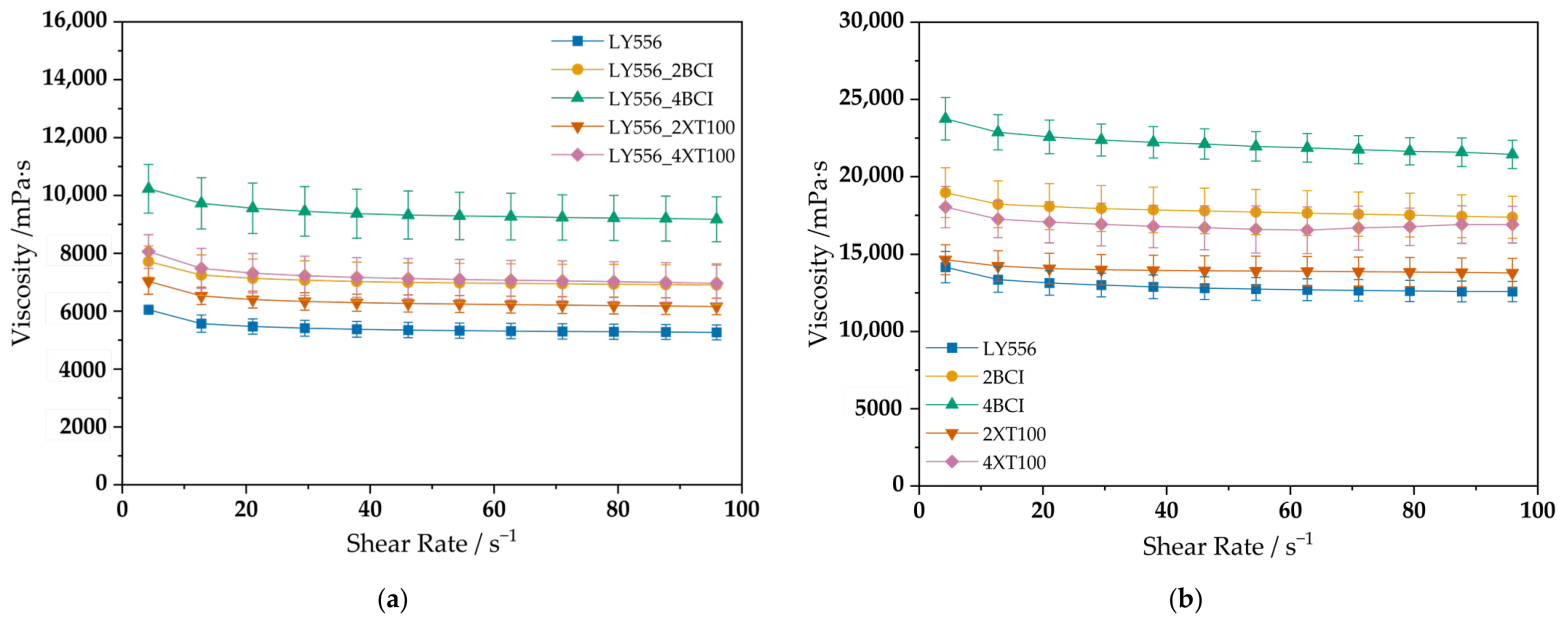
Viscosity versus the shear rate of the neat epoxy resin system and the modified counterparts: (**a**) after mixing (t = 0 min) and (**b**) after 2 h (t = 120 min).

Subsequently, rheological measurements were conducted to evaluate the processability of all the dispersions for the pre-impregnation equipment used. The viscosity of the samples was measured immediately after mixing the resin with hardeners and again at 120 min, which corresponds to the normal processing window. Figure 5 presents the results obtained.

The rheological results show that, at equivalent modifier loadings, BCI produces a greater increase in resin viscosity than Clearstrength^®^ XT100. This behaviour can be attributed to the distinct mechanisms by which the two modifiers interact with the epoxy matrix. Consequently, modifier type directly influences the practical impregnation window, with higher-viscosity BCI formulations reaching processing limits more rapidly than their Clearstrength^®^ XT100 counterparts. Another important observation is the substantial increase in viscosity across all formulations between t = 0 and t = 120 min, indicating early-stage resin advancement before cure. This reflects the resin’s ongoing reaction and effectively defines the usable working time for impregnation. In particular, the addition of 4 wt.% BCI, which starts from the highest baseline window, reaches unworkable viscosity levels first and therefore requires faster processing, whereas the 2 wt.% Clearstrength^®^ XT100 formulation retains a wider processing margin before viscosity becomes limiting. These results indicate that both modifier type and loading level are critical parameters governing available processing time and, consequently, the manufacturability of the resin system in pre-impregnated materials production.

Therefore, based on thermal stability, homogeneous dispersion, the highest Charpy impact results (suggesting increased toughness), and the best processability conditions, the 2 wt.% Clearstrength^®^ XT100 formulation was selected to produce pre-impregnated materials and evaluate its effects on the mechanical properties of CFRP laminates.

### 3.2. CFRP

Carbon pre-impregnated materials were produced to manufacture the CFRP laminates. Accordingly, their FVF was measured to ensure process consistency. The values obtained were 54 ± 3% and 50 ± 5% for the reference and modified pre-impregnated materials, respectively. The materials were then stacked and consolidated to produce the different laminates, with each ply having a nominal thickness of 0.1 mm.

**Figure 6:**
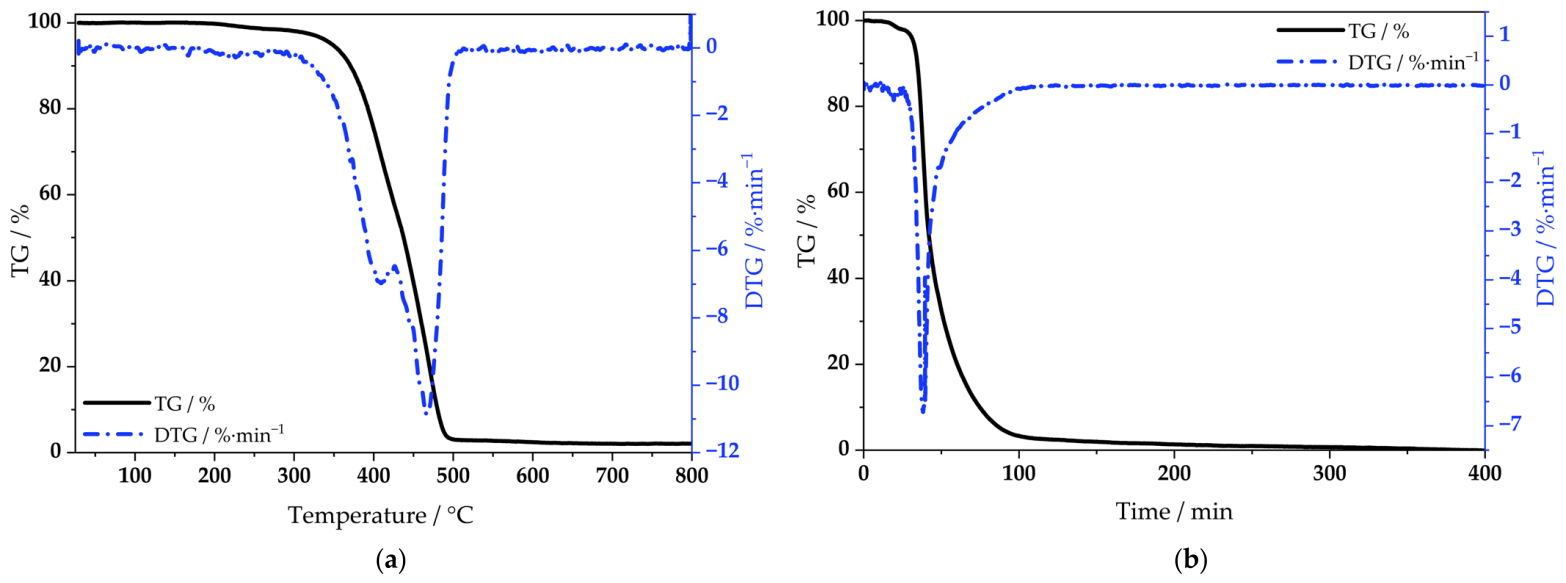
TGA results showing the thermogravimetric curve (black) and the derivative thermogravimetry curve (blue dotted line) of the Clearstrength^®^ XT100 particles. (**a**) Heating from 25 °C to 800 °C; (**b**) heating under the burn-off method.

TGA analyses of the particle Clearstrength^®^ XT100 were conducted before the determination of the FVF of the CFRP to evaluate their decomposition behaviour and determine whether it remained intact after the burning process or was degraded along with the resin. Figure 6 presents the TGA results where two different analyses were made: (i) the thermal behaviour of Clearstrength^®^ XT100 was examined when heated to 800 °C (Figure 6a), and (ii) the decomposition behaviour during the simulation of the burn-off method (6 h at 425 °C) of the composite material (Figure 6b). From the first analysis, in Figure 6a, it can be observed that, until 300 °C, the mass loss is negligible at approximately 3%, which can be attributed to impurities, humidity or plasticisers. The maximum degradation velocity is observed at 467 °C, while a preliminary stage of decomposition occurs at 409 °C, corresponding to the degradation of the methylmethacrylate, and the subsequent stage is linked to the breakdown of butadiene–styrene [19,37,38]. At the end of the test, only a 2% residue remained. From the simulation of the burn-off test procedure presented in Figure 6b, it was concluded that the residue of the particle encountered at the end of the test was negligible, since after 6 h, the TGA revealed no residue left, suggesting that the final mass remaining in the calcination crucible is only fibre.

After determining the degradation behaviour of Clearstrength^®^ XT100 particles, the FVF of both developed CFRPs (reference without any particle added (named Unmodified CFRP) and CFRP laminates modified with Clearstrength^®^ XT100 (named 2XT100 CFRP)) and density were determined. The fibre volume fraction is an important property to evaluate, as it represents the proportion of fibres within the CFRP and strongly influences the material’s mechanical properties. The composite density depends on the proportions and densities of the fibres and matrix, and the low density of CFRP contributes to its high strength-to-weight ratio. Together, fibre volume fraction and density are key indicators of composite quality and are widely used to assess manufacturing consistency and predict material performance. Therefore, the results for the density, fibre and resin volume fractions and void content are presented in Table 3.

Based on the results presented above, it can be concluded that incorporating Clearstrength^®^ XT100 particles did not change the density of the CFRP. Additionally, the fibre and resin fractions were held constant throughout the study to ensure comparability. As expected, the FVF increased in the CFRPs due to resin bleeding during autoclave consolidation. The void content remained low in both formulations (2.3 ± 1.5% and 1.3 ± 1.4% for the unmodified and 2XT100 CFRPs, respectively). However, the inherent uncertainty in the subtractive calculation of void content from fibre and resin volume fractions precludes a stronger claim of statistical equivalence between the two conditions.

Moreover, mechanical testing is essential for evaluating the performance of composite materials. When epoxy resin is modified, it is essential to ensure that the addition of particles does not compromise the composite’s mechanical properties, thereby maintaining the structure’s structural integrity. In this study, tensile tests in both longitudinal and transverse directions and Mode I interlaminar fracture toughness tests were conducted.

Tensile testing determines the composite’s strength, stiffness, and deformation behaviour under axial loading. Tensile tests in the longitudinal and transverse directions of CFRPs provide complementary information on the composite’s anisotropic behaviour. Longitudinal tensile testing evaluates properties dominated by the carbon fibres, typically yielding high strength and stiffness. In contrast, transverse tensile testing primarily assesses the matrix and fibre–matrix interface, which exhibit significantly lower mechanical properties. Combining both directions is essential for understanding the load-bearing capabilities and failure mechanisms of CFRP laminates. In Table 4, the tensile results in the longitudinal direction at RT and CT are presented.

Table 4 indicates that incorporating 2 wt.% of Clearstrength^®^ XT100 had limited influence on the fibre-dominated mechanical response of the laminates. At RT, the tensile strength decreased from 2043 ± 106 MPa for the unmodified CFRP to 1924 ± 186 MPa for the modified laminate, corresponding to a reduction of approximately 6%. However, the relatively large standard deviation observed suggests that this decrease may not be statistically significant. A Student’s *t*-test assuming equal variances was performed to confirm this observation, revealing no statistically significant difference between the two groups (t(8) = 1.233, *p* = 0.253). At CT, the modified laminate exhibited a slight increase in tensile strength, from 1904 ± 64 MPa to 1942 ± 51 MPa, representing an improvement of about 2%; however, this increase was not statistically significant (*p* = 0.330). Therefore, these results indicate that the addition of Clearstrength^®^ XT100 does not compromise the tensile load-bearing capability. A similar trend was observed for Young’s modulus, where at RT, the modulus decreased slightly from 135 ± 3 GPa to 129 ± 5 GPa following modification. Although this difference approached statistical significance (t(8) = 2.234, *p* = 0.056), it did not reach the conventional threshold (*p* < 0.05). At CT, both materials exhibited comparable average modulus values of approximately 122 GPa. Overall, the modification had a limited effect on Young’s modulus under both test conditions.

Considering the relatively small differences between the measured values and the statistical analysis performed, it can be concluded that the incorporation of 2 wt.% Clearstrength^®^ XT100 does not significantly affect the longitudinal tensile properties of the CFRP laminates. This behaviour is expected, since the carbon fibres primarily govern the tensile response in the fibre direction, whereas the toughening agent acts mainly on the polymer matrix and the fibre–matrix interfacial region.

For this, the tensile results from transversal tests are presented in Table 5 for both laminates and testing conditions.

In contrast to the longitudinal tensile results, the transverse properties exhibited a noticeable improvement following matrix modification, indicating a significant influence of the toughening agent on the matrix-dominated mechanical behaviour of the composites. At RT, the transverse tensile strength increased from 30 ± 1 MPa for the unmodified CFRP to 35 ± 4 MPa for the modified laminate, corresponding to a statistically significant improvement of approximately 17% (*p* = 0.039). An even greater enhancement was observed at cryogenic temperature, where the tensile strength increased from 29 ± 3 MPa to 37 ± 6 MPa, representing a statistically significant gain of nearly 28% (*p* = 0.029). These results suggest that incorporating Clearstrength^®^ XT100 improves load transfer within the matrix and enhances the resistance of the fibre–matrix interface to crack initiation and propagation, particularly under cryogenic conditions. At CT, the arrangement of the resin molecules tightens, leading to stronger intermolecular bonding and, consequently, a higher tensile strength [21]. On the other hand, at RT, both laminates exhibited similar Young’s modulus values of approximately 7 GPa, indicating that the addition of the toughening agent did not significantly affect the transverse stiffness. At CT, the unmodified laminate exhibited a slightly higher modulus of 8 GPa, whereas the modified laminate maintained 7 GPa, a statistically significant difference (*p* = 0.004).

Overall, the transverse tensile results demonstrate that the addition of 2 wt.% Clear-strength^®^ XT100 effectively enhances the matrix-controlled mechanical performance of the CFRP laminates, with the benefits becoming more pronounced under cryogenic conditions. The improved transverse strength, combined with the negligible changes in Young’s modulus at RT, indicates that the toughening agent enhances resistance to matrix cracking and interfacial failure without compromising the composite’s inherent stiffness at RT. At CT, however, the modest yet statistically significant reduction in modulus suggests a slight trade-off between strength and stiffness.

**Figure 7:**
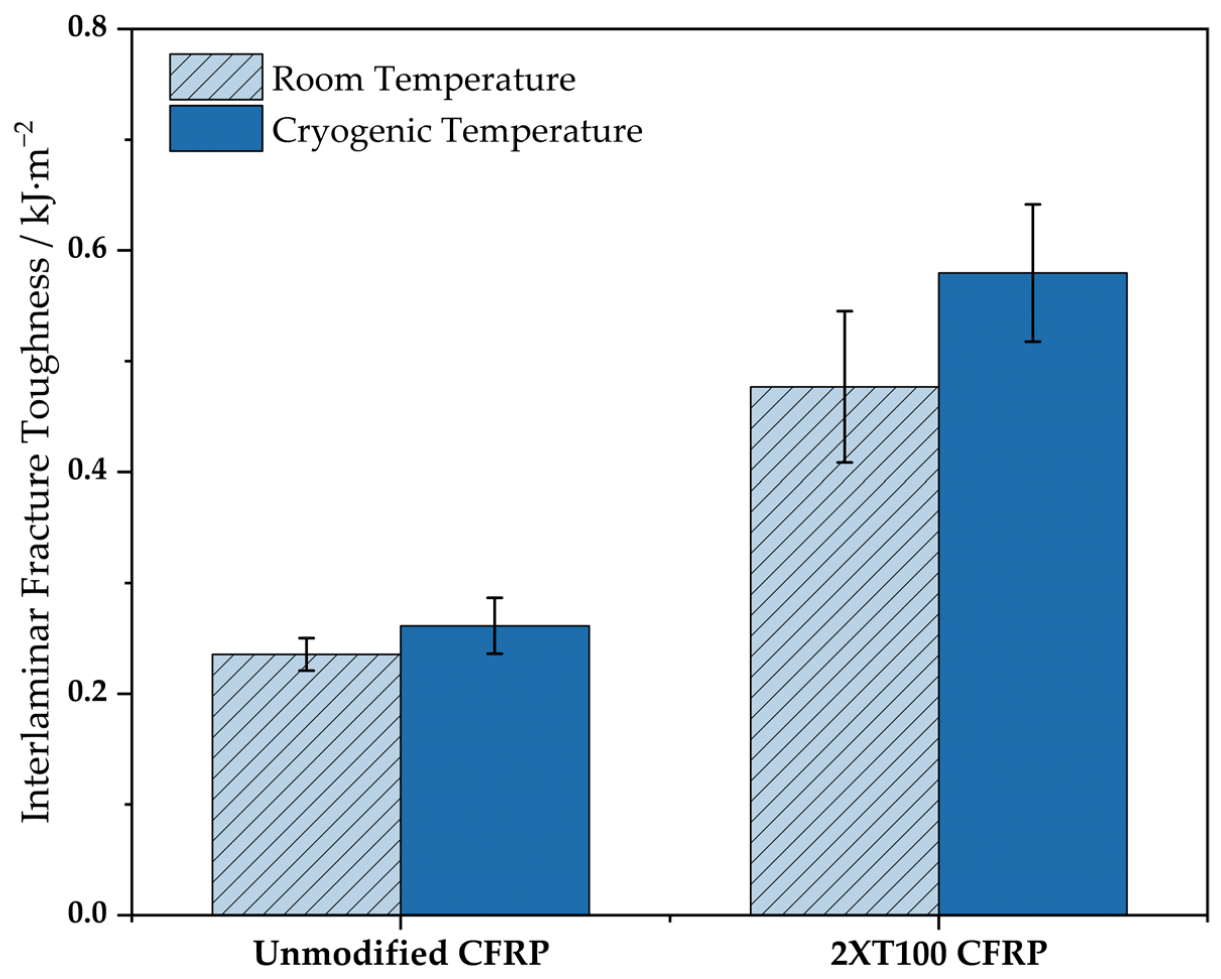
Interlaminar fracture toughness (G_IC_) results.

Finally, to investigate the study’s target property, Mode I interlaminar fracture toughness was evaluated using DCB tests. The main results at RT and CT are presented in Figure 7.

In both conditions, the modified laminates exhibited higher G_IC_ values than the unmodified CFRP, indicating improved interfacial bonding and resistance to interlaminar failure. The addition of 2 wt.% of the MBS core–shell to the matrix increased the interlaminar fracture toughness of the CFRP from 0.24 to 0.48 kJ·m^−2^ at RT, which represents a highly statistically significant increase of 102% (*p* = 0.00006). This result suggests an enhancement of load transfer between the fibre and matrix phases. This magnitude of improvement is consistent with previous reports on CSR-toughened CFRP, where room-temperature G_IC_ gains typically range from approximately 50% to 130%, depending on the CSR type, particle content, and toughening mechanism involved [19,35,39,40]. Additionally, similar results have been reported in the literature for CFRP reinforced with the same particle. As mentioned in previous sections, for instance, Beylergil et al. [19] reported that adding 3 wt.% of Clearstrength^®^ XT100 increased G_IC_ by 70% compared to unmodified CFRP. Higher concentrations of 5 wt.% and 7 wt.% resulted in improvements of 91% and 177%, respectively. A more pronounced improvement was observed under cryogenic conditions, where the modified laminate achieved the highest G_IC_ values, increasing from 0.26 to 0.58 kJ·m^−2^ compared to the unmodified CFRP, corresponding to an improvement of 122%, (*p* < 0.001).

In particular, the unmodified CFRP showed only a marginal and statistically non-significant increase in G_IC_ between RT and CT (0.24 to 0.26 kJ·m^−2^, +8%, *p* = 0.084), whereas the modified laminate exhibited a substantially larger absolute gain over the same temperature range (0.48 to 0.58 kJ·m^−2^, +21%, *p* = 0.038). This indicates that the higher relative gain at low temperatures is not simply a consequence of a smaller reference value but reflects a more efficient contribution of MBS particles to interlaminar toughening during cryogenic cycling. This behaviour may be attributed to partial debonding of MBS core–shell particles from the epoxy matrix during cryogenic cycling, driven by a mismatch in the thermal expansion coefficients (CTEs) between the particles and the surrounding epoxy resin. Such debonding is widely recognised as an initiating step in the fracture of rubber-toughened epoxies, promoting subsequent matrix shear yielding and increased energy dissipation during crack propagation [41,42,43]. Although direct evidence of particle debonding was not obtained in the present study, the marked increase in G_IC_ observed only in the modified laminate is consistent with this mechanism and with previous reports on CSR-modified epoxy systems at low temperatures [36]. This interpretation is further supported by the unmodified laminate, which has no particles to debond and shows almost no change between RT and CT.

In the present work, despite the use of a lower particle concentration, i.e., 2 wt.%, very promising results were achieved, with improvements exceeding those reported for 5 wt.% and approaching values reported in the literature for 7 wt.% incorporations [19]. Furthermore, the material exhibited consistent improvements under both ambient and cryogenic conditions, highlighting the effectiveness and stability of the proposed modification strategy and the potential for use in applications requiring high structural reliability at low temperatures.

**Figure 8:**
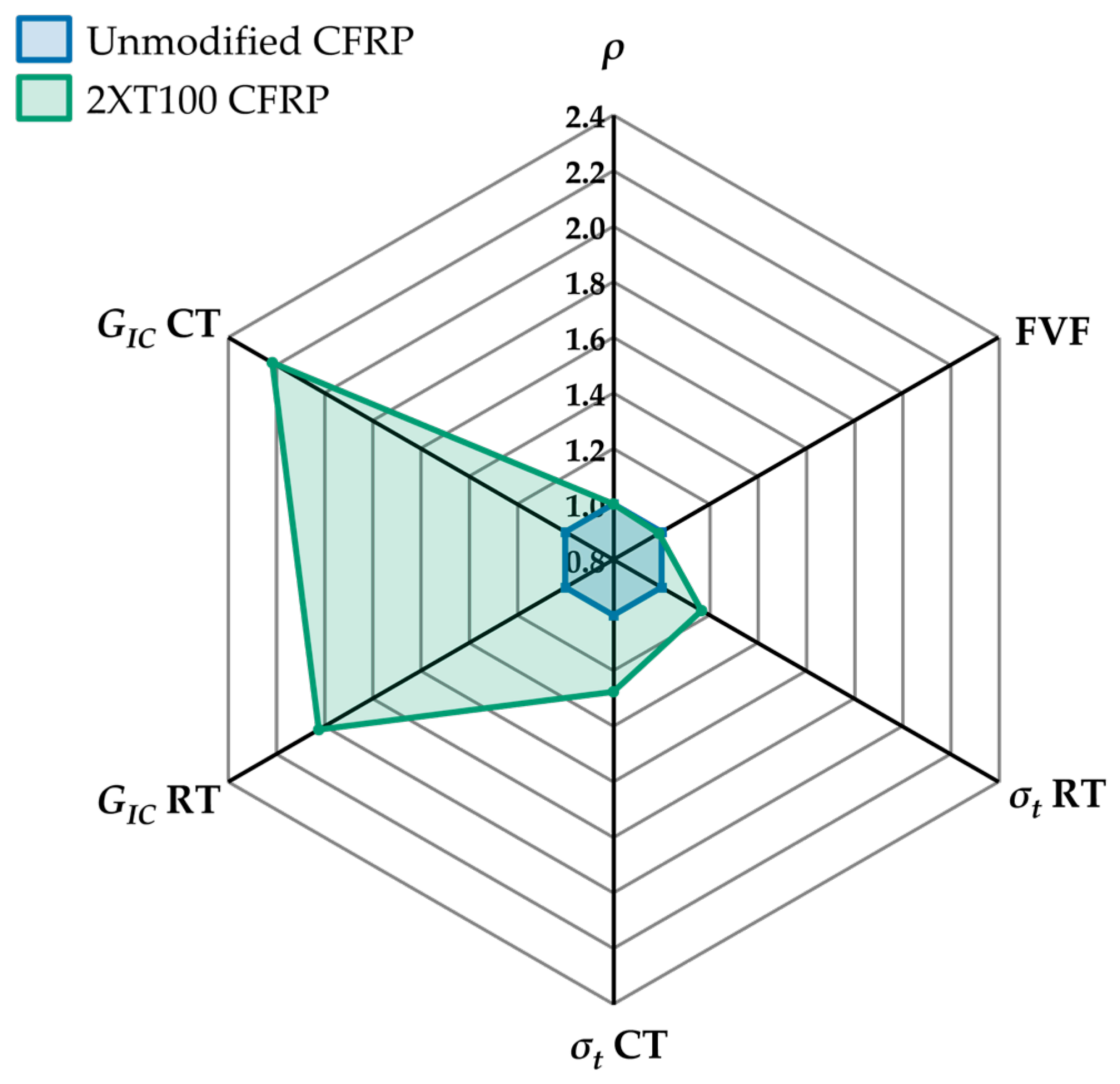
Comparative radar plot of the mechanical properties of the CFRP under study at RT and CT. *ρ* denotes the density, FVF the fibre volume fraction, *σ_T_* the tensile strength in the transverse direction and *G_IC_* the interlaminar fracture toughness mode I. All values are normalised to the unmodified CFRP.

Figure 8 presents a radar plot summarising the target properties normalised to the reference CFRP.

Values that extend further from the centre indicate improvements in relation to the baseline (unmodified CFRP). From the plot, the modified CFRP exhibited improved performance at both RT and CT, with higher transverse mechanical properties and a significant increase in interlaminar toughness, indicating substantial strength enhancements following matrix modification. Together, these findings indicate that the matrix modification process with a low content of Clearstrength^®^ XT100 primarily improves matrix-dominated and interfacial properties, while having a negligible influence on fibre-dominated longitudinal tensile behaviour.

## 4. Conclusions

In this work, the first study evaluated the incorporation of two tougheners from different categories into nanocomposites. It was found that the optimal additive and concentration were 2 wt.% of Clearstrength^®^ XT100. This formulation was subsequently used to produce CFRP laminates, which were evaluated using longitudinal and transverse tensile tests and interlaminar fracture toughness measurements. Based on this investigation, the following conclusions can be drawn:

- For nanocomposites, the addition of 2 wt.% of Clearstrength^®^ XT100 increased the absorbed energy by 125% compared with the neat epoxy resin. By contrast, the addition of the BCI resin did not significantly affect the properties of the nanocomposite.
- The addition of a selected particle to CFRP does not influence fibre and resin volume fractions of the composite laminate.
- The incorporation of Clearstrength^®^ XT100 did not significantly affect the longitudinal tensile properties, tensile strength or modulus in the longitudinal direction, at either RT or CT.
- The transverse tensile strength increased by 28% at cryogenic temperature when core–shell particles were added.
- The interlaminar fracture toughness of the CFRP increases by 102% and 122% with the addition of 2 wt.% of Clearstrength^®^ XT100 in relation to the unmodified counterpart for RT and CT, respectively.

The results of this study indicate that adding various tougheners may significantly improve the performance of CFRP under thermal cycling, contributing to the development of materials for high-performance applications.

Future work includes studying different types of additives, such as ceramic or block copolymer materials, conducting comprehensive characterisation, and using an improved methodology for cryogenic cycling with a broader number of cryogenic cycles to identify possible damage saturation or plateau regions.

## Figures and Tables

**Table 1:** Particle concentration studied and respective incorporation method.

Particle	Nomenclature	Studied Concentrations	Method of Incorporation
Biscitraconimide-based resin (BCI)	LY556_2BCI	2 wt.%	Mechanical stirrer with silicone bath heating
	LY556_4BCI	4 wt.%	
Clearstrength^®^ XT100	LY556_2XT100	2 wt.%	
	LY556_4XT100	4 wt.%	

**Table 2:** Glass transition temperature results.

Sample	Glass Transition Temperature	Variation in Relation to the Neat Epoxy
LY556	114.8 ± 0.5	
LY556_2BCI	110.4 ± 0.5	−4%
LY556_4BCI	115.1 ± 1.5	≈
LY556_2XT100	109.2 ± 0.9	−5%
LY556_4XT100	110.9 ± 0.3	−3%

**Table 3:** Density, fibre and resin volume fractions and void content results.

Sample	Density/g·cm^−3^	Fibre Volume Fraction/%	Resin Volume Fraction/%	Void Content/%
Unmodified CFRP	1.50 ± 0.04	55.8 ± 3.2	41.9 ± 2.1	2.3 ± 1.5
2XT100 CFRP	1.50 ± 0.02	55.2 ± 2.3	43.6 ± 2.7 *	1.3 ± 1.4

* Corresponds to the resin modified with the Clearstrength^®^ XT100, including the particle.

**Table 4:** Results for the longitudinal tensile tests (0°).

Sample	Tensile Strength/MPa		Young’s Modulus/GPa	
	RT	CT	RT	CT
Unmodified CFRP	2043 ± 106	1904 ± 64	135 ± 3	122 ± 2
2XT100 CFRP	1924 ± 186	1942 ± 51	129 ± 5	122 ± 3

**Table 5:** Results for the transversal tensile tests (90°).

Sample	Tensile Strength/MPa		Young’s Modulus/GPa	
	RT	CT	RT	CT
Unmodified CFRP	30 ± 1	29 ± 3	7.0 ± 0.2	8.0 ± 0.2
2XT100 CFRP	35 ± 4	37 ± 6	7.0 ± 0.2	7.2 ± 0.4

## Data Availability

The original contributions presented in this study are included in the article. Further inquiries can be directed to the corresponding authors.

## Abbreviations

The following abbreviations are used in this manuscript:

ANOVA	Analysis of Variance
BCI	Biscitraconimide
BSEA	Phosphorus-/Nitrogen-Containing Fire Retardant
CFRPs	Carbon Fibre-Reinforced Polymers
CNT	Carbon Nanotubes
CSR	Core–Shell Rubber
CT	Cryogenic Temperature
CTE	Coefficient of Thermal Expansion
DCB	Double Cantilever Beam
DSC	Differential Scanning Calorimetry
FRP	Fibre-Reinforced Polymers
FVF	Fibre Volume Fraction
GNP	Graphene Nanoplatelet
MBS	Methylmethacrylate–Butadiene–Styrene
MBT	Modified Beam Theory
PTFE	Polytetrafluoroethylene
RT	Room Temperature
SEM	Scanning Electron Microscopy
*T_g_*	Glass Transition Temperature
TGA	Thermogravimetric Analyses
